# When Irrigation Flows the Wrong Way: Intraoperative Regurgitation Through an Enterovesical Fistula

**DOI:** 10.7759/cureus.117318

**Published:** 2026-10-01

**Authors:** Leonardo Monteiro, João V Pais, Rui F Santos, Anabela S Vieira, Sara Mota

**Affiliations:** 1 Anesthesiology, Unidade Local de Saúde do Tâmega e Sousa, Penafiel, PRT

**Keywords:** airway management, cystolithotripsy, enterovesical fistula, ileal pouch-anal anastomosis, inflammatory bowel disease, intraoperative regurgitation, irrigation fluid

## Abstract

Enterovesical fistulas complicate inflammatory bowel disease, particularly after restorative proctocolectomy with ileal pouch-anal anastomosis, creating pathways that may complicate endoscopic urological procedures performed under continuous irrigation.

A 33-year-old man (61 kg; body mass index 18.8 kg/m²) with unclassified pancolitis (no histological features of Crohn disease in the colectomy specimen) had undergone restorative proctocolectomy with an ileal J-pouch, defunctioning loop ileostomy and ileal pouch-anal anastomosis, with repeated failed interventions for a chronic enterovesical fistula. He underwent elective laser cystolithotripsy and suprapubic catheter exchange. After induction with fentanyl, lidocaine, propofol and rocuronium, the airway was managed with a size 4 i-gel (Intersurgical Ltd, Wokingham, UK), without an orogastric tube in its gastric channel, in the lithotomy position, with gravity-fed 0.9% saline irrigation.

One hour and 23 minutes after the start of surgery, clear fluid appeared at the oropharynx. The oropharynx was suctioned, the trachea was intubated, and a nasogastric tube was placed; a second episode followed two minutes later. Irrigation was stopped. The ileostomy appliance, empty at induction, was completely distended with clear fluid, and irrigation return through the cystostomy was markedly reduced. Core temperature on admission to the intermediate care unit was 33.0 °C. Ventilatory parameters, auscultation, arterial blood gas analysis, and chest radiography showed no evidence of aspiration. He was discharged on postoperative day 7.

We propose that the regurgitated fluid was predominantly irrigation fluid that had migrated retrogradely through the fistula into the ileal pouch and small bowel. To our knowledge, this mechanism has not been described previously. Fistulous urinary-gastrointestinal anatomy warrants treatment as an aspiration risk factor in its own right, with gastric-channel drainage whenever a supraglottic device is used, and deliberate monitoring of irrigation input, drainage return, stomal output, and core temperature.

## Introduction

An enterovesical fistula is an abnormal communication between the gastrointestinal tract and the urinary bladder. Most arise from diverticular disease, colorectal or pelvic malignancy, or Crohn disease, and a smaller proportion follow pelvic surgery or radiotherapy; ulcerative colitis is an uncommon cause [1,2]. The characteristic clinical features are pneumaturia, fecaluria, and recurrent or polymicrobial urinary tract infections, although the presentation may remain indolent for years [1,3]. After restorative proctocolectomy with ileal pouch-anal anastomosis, fistulas involving the pouch are well recognized, with reported rates of pouch-related fistulas ranging from 5% to 13%, although fistulas specifically involving the bladder remain rare [4,5].

Endoscopic cystolithotripsy is routinely performed under continuous irrigation with isotonic solutions at relatively low intravesical pressures. In the absence of bladder perforation, significant extravesical spread of irrigation fluid is uncommon [6,7]. An intravesical pressure of only about 10 cm H₂O may nonetheless suffice to drive fluid absorption through exposed venous sinuses [6]. In patients with chronic enterovesical fistulas, however, the bladder is structurally continuous with the bowel, and the physiological barrier that normally contains irrigation fluid is absent. A sustained hydrostatic gradient may therefore drive fluid along a preformed conduit into the gastrointestinal lumen. Whether such fluid then progresses proximally, against the normal direction of peristalsis, depends on additional conditions, most of them characteristic of general anesthesia rather than of the fistula itself: pharmacologically suppressed propulsive activity, neuromuscular blockade, a compliant proximal reservoir, and time [8]. These conditions, and their convergence in a single patient, are the substance of this report.

Intraoperative regurgitation and pulmonary aspiration remain a leading cause of anesthesia-related morbidity and mortality. In the 4th National Audit Project (NAP4) of the Royal College of Anaesthetists and the Difficult Airway Society, 133 major airway complications were reported during anesthesia, resulting in 16 deaths; aspiration was the single most common primary event leading to death, accounting for approximately half of these deaths, and inappropriate selection or use of supraglottic airway devices was a recurrent contributory theme [9]. Supraglottic airway devices are used in the majority of general anesthetics in the United Kingdom and are frequently selected for elective endourological procedures, but their use in patients with anatomical abnormalities of the gastrointestinal tract deserves careful consideration [10,11].

We report a case of intraoperative regurgitation during elective cystolithotripsy in a patient with a longstanding enterovesical fistula, in which the regurgitated fluid was interpreted as cystoscopic irrigation fluid that had migrated retrogradely through the fistula into the ileal pouch and small bowel. To our knowledge, this mechanism of intraoperative regurgitation has not previously been described in the published literature. The report also addresses two questions that existing guidance does not cover: how such an event can be recognized early, and how the airway should be managed when fistulous anatomy is known or suspected.

## Case presentation

Background and previous surgical history

A 33-year-old man was admitted for elective laser cystolithotripsy and replacement of a chronic suprapubic cystostomy. His past medical history was notable for pancolitis, first diagnosed in 2018 and never definitively classified (the colectomy specimen showed no histological features of Crohn disease), for which he had undergone total proctocolectomy with terminal ileostomy, followed by laparoscopic construction of an ileal J-pouch and, several months later, a combined procedure comprising defunctioning loop ileostomy, mucosectomy of the rectal stump, ileal pouch-anal anastomosis, and intraoperative identification of an enterovesical fistula. A subsequent urological attempt at transvesical closure of the fistula, accompanied by bilateral double-J ureteric stenting, was unsuccessful. He was later admitted for pyelonephritis associated with the persistent fistula, after which a suprapubic cystostomy was placed. This was subsequently replaced during a complete endoscopic cystolithotripsy performed for stone-related catheter obstruction, and a further admission for orchiepididymitis treated with piperacillin/tazobactam occurred before the index admission.

In the months preceding the index procedure, his fistula-related symptoms comprised occasional loss of urine through the ileostomy and loss of fluid through the anus on straining; no pneumaturia, fecaluria, or recurrent urinary tract infection was recorded during that period. The patient took no regular medication and reported no known drug allergies; he identified a prior intolerance to ibuprofen in the context of colitis activity.

Preoperative assessment

The patient's weight was 61 kg, height 1.80 m, and body mass index 18.8 kg/m², consistent with a lean habitus at the lower limit of the normal range. Preoperative airway examination disclosed a modified Mallampati class II oropharynx with normal thyromental distance and normal cervical mobility. Preoperative fasting was eight hours for solids and two hours for clear fluids, in accordance with institutional protocol. Preoperative computed tomography of the abdomen and pelvis demonstrated the indwelling suprapubic catheter and multiple intravesical calculi (Figure 1A), together with a defect in the posterior bladder wall corresponding to the fistula (Figure 1B).

**Figure 1:**
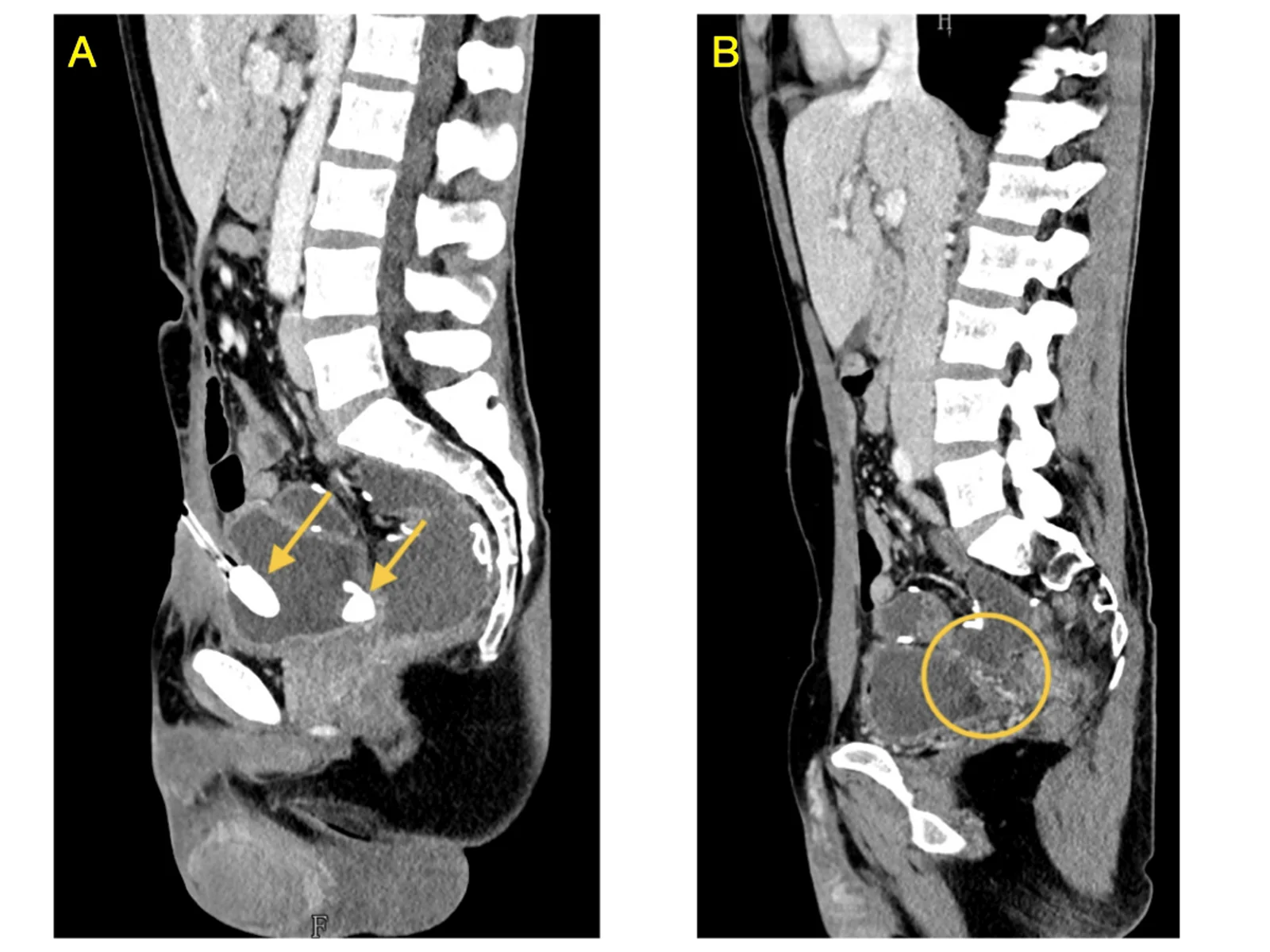
Preoperative computed tomography of the abdomen and pelvis (sagittal reconstructions). (A) The indwelling suprapubic catheter (left arrow) and an intravesical calculus (right arrow) are indicated. (B) The circle indicates the defect in the posterior bladder wall corresponding to the enterovesical fistula.

Taking into account the patient's low body mass index, a chronic enterovesical fistula with continuous enteric contamination of the urinary tract, multiple previous major abdominal operations, and recurrent urinary and systemic infections, the patient was classified as American Society of Anesthesiologists (ASA) physical status 3 [12].

Anesthetic technique

Standard monitoring included five-lead electrocardiography, pulse oximetry, noninvasive blood pressure measurement, capnography, end-tidal gas analysis, bispectral index (BIS), and quantitative neuromuscular monitoring. General anesthesia was induced at 14:54 with intravenous fentanyl 150 μg (2.5 μg/kg), lidocaine 50 mg (0.8 mg/kg), propofol 100 mg (1.6 mg/kg), and rocuronium 20 mg (0.3 mg/kg).

A size 4 i-gel supraglottic airway (Intersurgical Ltd, Wokingham, UK) was placed at the first attempt. An adequate seal was confirmed by the absence of an audible leak, a square-wave capnogram, and a stable expired tidal volume. No orogastric tube was passed through the gastric channel of the device at induction. The manufacturer recommends the size 4 device for patients weighing approximately 50-90 kg; at 61 kg, the patient lay within this range and above the 30- 60 kg range of the size 3.

Anesthesia was maintained with sevoflurane at an end-tidal concentration of 1% to 2%, titrated to a BIS value of 40 to 60. Ventilation was pressure-controlled, with peak airway pressures of 11 to 13 cm H₂O, positive end-expiratory pressure of 5 cm H₂O, and end-tidal carbon dioxide of 30 to 35 mm Hg. Cefoxitin 2 g was administered for antibiotic prophylaxis. Dexamethasone 4 mg was given for prophylaxis of postoperative nausea and vomiting (Apfel score of 3: non-smoker, previous postoperative nausea and vomiting, and postoperative opioid use). Active warming consisted of a forced-air warming blanket, and intravenous fluids were not warmed. The patient was placed in the lithotomy position.

Surgical procedure and irrigation set-up

The surgical procedure began shortly after induction. Urethrocystoscopy revealed an encrusted suprapubic catheter surrounded by a calculus of approximately 2 cm, together with two additional intravesical calculi of similar size. An epithelialized fistulous tract approximately 2 cm in diameter was identified in the posterior bladder wall. Complete laser cystolithotripsy was performed with continuous normal saline irrigation delivered by gravity from a bag suspended 40 cm above the level of the bladder, corresponding to a nominal hydrostatic pressure of approximately 40 cm H₂O. The irrigation fluid was at ambient theatre temperature, approximately 20-22 °C, and the total irrigation volume was not quantified. The total surgical duration was 1 hour and 50 minutes, and total anesthesia time was two hours and two minutes. No intraoperative endoscopic images were recorded.

Intraoperative event and immediate management

One hour and 23 minutes after the start of the procedure (16:25), an episode of clear-fluid regurgitation was noted at the i-gel. The supraglottic airway was removed, direct oropharyngeal suctioning was performed under laryngoscopic vision, and the trachea was intubated on the first attempt with a cuffed 7.0 mm tube (Cormack-Lehane grade III view with a size 4 Macintosh blade); before ventilation was resumed, the trachea was suctioned through the tube; the aspirate was unremarkable, with no gastric or particulate content recovered. Correct placement was confirmed by capnography and auscultation. A 14-French nasogastric tube was placed and connected to continuous low-pressure suction. Approximately two minutes later, a second episode of clear fluid was observed draining through the nasogastric tube; 400 mL were recovered in total.

At this point, the ileostomy appliance was inspected for the first time since the start of surgery. It had been emptied and was flat at induction and lay beneath the surgical drapes throughout; it was now completely distended with clear fluid, with an estimated volume of 750 mL. Simultaneously, the return of irrigation effluent through the cystostomy was markedly reduced. Irrigation was stopped immediately. These observations, taken together, supported the interpretation that irrigation fluid used during cystolithotripsy had migrated retrogradely through the enterovesical fistula into the ileal pouch and small bowel, with progressive filling of the proximal gut and subsequent regurgitation through the upper gastrointestinal tract (Figure 2).

**Figure 2:**
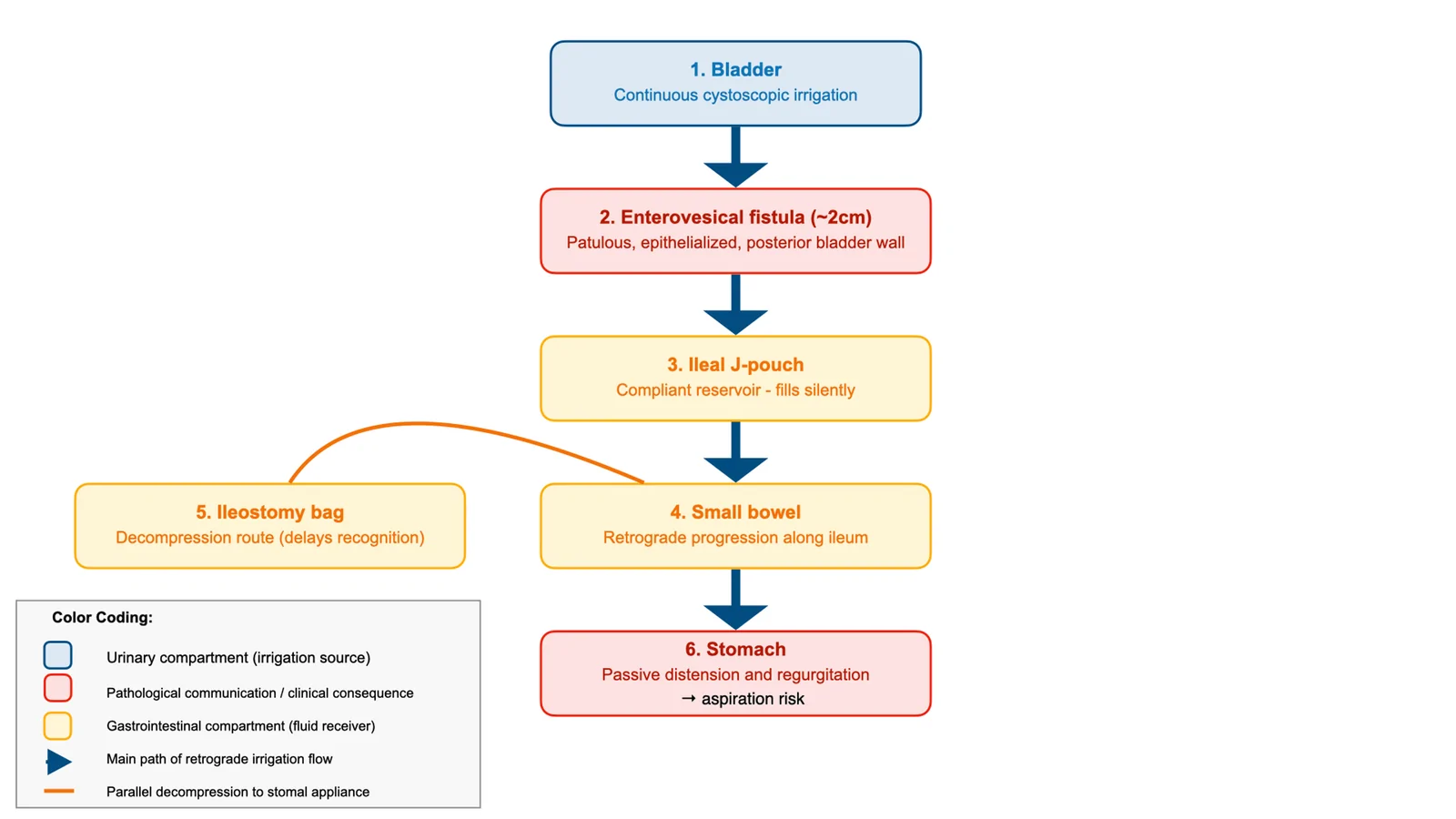
Schematic representation of the proposed pathway of retrograde fluid migration. Continuous saline irrigation introduced through the cystoscope into the bladder (1) exits through the chronic enterovesical fistula (2) into the ileal J-pouch (3). Fluid then ascends retrogradely along the small bowel (4), partly decompressing through the defunctioning loop ileostomy and filling the stoma appliance (5), and partly progressing proximally to distend the stomach, ultimately producing passive regurgitation (6).

Auscultation of the chest after intubation was symmetric, with normal breath sounds and no adventitious sounds. Airway pressures, tidal volumes, end-tidal carbon dioxide, and oxygen saturation remained within the pre-event range for the remainder of the procedure. Intraoperative abdominal ultrasonography performed by the urologist did not identify free intraperitoneal fluid or any other abnormality. The cystostomy was exchanged for a 16-Frenchsilicone catheter, and a 20-French silicone urethral catheter was placed for additional bladder drainage. Neuromuscular blockade was reversed with sugammadex 150 mg (2.5 mg/kg). Hydrocortisone 200 mg was administered after the event, given empirically because pulmonary aspiration was suspected and bronchospasm was considered a risk. Postoperative analgesia included intravenous paracetamol 1,000 mg and tramadol 100 mg; ondansetron 8 mg was administered, providing dual antiemetic prophylaxis together with the dexamethasone given at induction.

Given the recognized risk of occult pulmonary aspiration and the need to monitor systemic response to possible fluid absorption, the patient was transferred to the intermediate care unit for overnight observation rather than to the post-anesthesia care unit.

Postoperative course

On admission to the intermediate care unit, the patient was awake, cooperative, and oriented. Vital signs were blood pressure 113/85 mm Hg, heart rate 90 beats/min, respiratory rate 10 breaths/min, oxygen saturation 99% on 2 L/min by nasal cannula, and core temperature 33.0 °C. Active rewarming with forced-air warming was instituted; the time taken to reach 36.0 °C or above was not recorded.

An arterial catheter was placed in the right radial artery. An arterial blood gas on 2 L/min oxygen by nasal cannula showed pH 7.33, pCO₂ 45 mm Hg, pO₂ 120 mm Hg, SaO₂ 98%, HCO₃⁻ 23.7 mmol/L, ionized calcium 1.09 mmol/L (reference range: 1.15-1.29 mmol/L), and lactate 0.4 mmol/L (reference range: 0.5-1.6 mmol/L) - a mild, predominantly respiratory acidosis with normal oxygenation, a normal bicarbonate, and a normal lactate, consistent with residual anesthetic effect and hypothermia rather than with pulmonary injury, hypoperfusion, or a large hyperchloremic load. At 24 hours, serum sodium was 131 mmol/L and serum chloride 93 mmol/L, compared with preoperative values of 138 and 105 mmol/L, respectively.

Hemoglobin decreased from 124 g/L to 117 g/L (reference range: 130-170 g/L), leukocyte count rose from 5.7 × 10⁹/L to 16.6 × 10⁹/L (reference range: 4.5-11.0 × 10⁹/L), and C-reactive protein (reference range: <5.0 mg/L) rose from 10 mg/L to 280 mg/L over the first 24 hours; C-reactive protein subsequently fell to 120 mg/L at 72 hours. This trajectory was interpreted as a postoperative systemic inflammatory response rather than as established infection. Urinalysis obtained on the first postoperative day showed proteinuria, hematuria, and leukocyturia; these findings were anticipated in a patient with a chronic enterovesical fistula and an indwelling suprapubic catheter and, in the absence of fever, were not taken as evidence of new infection.

Chest radiography on the first postoperative day showed no acute pleuropulmonary abnormalities. The arterial blood gas the following morning had normalized (pH 7.43, pO₂ 86 mm Hg, pCO₂ 42 mm Hg, HCO₃⁻ 27.4 mmol/L, SaO₂ 97%). The patient remained afebrile throughout, and blood and urine cultures obtained before transfer - taken because of the chronic fistulous contamination of the urinary tract and the instrumentation of a colonized, stone-bearing bladder, rather than pre-emptively for aspiration - ultimately returned negative; no antimicrobial therapy beyond surgical prophylaxis was initiated. The patient had an uneventful 24-hour stay in the intermediate care unit. He was transferred to the urology ward and discharged home on postoperative day 7.

Follow-up

At outpatient follow-up three months later, the symptoms of fistula patency (spontaneous urination with continued loss of urine through the ileostomy, particularly at night, and loss of fluid through the anus with abdominal straining) were unchanged from the preoperative pattern. He declined further surgical intervention. At a further follow-up approximately 14 months after the procedure, he remained satisfied with his functional status, had not experienced further urinary tract infections, and a repeat renal ultrasound showed no hydronephrosis and no lithiasis.

## Discussion

This case illustrates an unusual mechanism of intraoperative regurgitation during an otherwise routine endoscopic urological procedure. We propose that irrigation fluid migrated retrogradely through a chronic enterovesical fistula into the ileal J-pouch and small bowel, filled the ileostomy appliance and the proximal gut, and ultimately distended the stomach, producing passive regurgitation (Figure 2). The explanation rests on clinical inference rather than direct measurement and is offered as the most parsimonious account of the observations.

Mechanism of retrograde migration

Enterovesical fistulas create a direct communication between structures that are normally separated by intact bladder and bowel walls [1,2]. The tract in this case was epithelialized and approximately 2 cm in diameter. We are not aware of data quantifying flow resistance across such a tract; that it offered little resistance is an inference from the Hagen-Poiseuille relationship, in which resistance varies inversely with the fourth power of the radius, and from the bidirectional flow observed in the awake patient. Fluid absorption syndromes during endourological procedures are classically described in the context of transurethral resection of the prostate and, more recently, of holmium laser enucleation of the prostate, where irrigation fluid enters the systemic circulation through exposed venous sinuses or extravesical spaces [6,13,14]. Intraperitoneal extravasation of irrigation fluid following bladder perforation has also been reported as a cause of postoperative abdominal distension and respiratory compromise [7]. The present case illustrates a third pathway: flow along a preformed conduit into the lumen of the gastrointestinal tract.

The most striking step is proximal progression to the stomach, against the normal direction of peristalsis, and several factors plausibly converged. General anesthesia and opioids suppress propulsive motility [8], so that luminal contents move along pressure gradients rather than by peristalsis, and gravity-fed irrigation sustained such a gradient for almost an hour and a half. An intravesical pressure as low as 10 cm H₂O may suffice to drive fluid absorption through exposed venous sinuses during endourological procedures [6]. Here the bag hung 40 cm above the bladder, well below the 60 cm conventionally recommended [6,13], and the event occurred regardless. The conventional ceiling is calibrated against absorption into venous sinuses, whereas a wide fistula draining to bowel decompressed to atmospheric pressure transmits flow at any positive gradient; limiting the height reduces the rate of migration rather than preventing it. Neuromuscular blockade abolished abdominal wall tone, and the lithotomy position reduced the gravitational disadvantage of proximal flow. The J-pouch accommodated volume at low pressure and filled silently. A loop ileostomy is not divided: once the appliance was full, both stomal openings were submerged, the decompressive route was lost, and pressure could be transmitted proximally along the afferent limb. Finally, loss of fluid through the anus on straining, documented before surgery, indicates a patent pouch-anal anastomosis; with the sphincter relaxed in lithotomy, some fluid probably escaped unrecognized into the drapes, which may partly explain why the discrepancy between irrigation input and return was not appreciated earlier.

Systemic absorption

Classical transurethral resection syndrome results from intravascular absorption of hypotonic, electrolyte-free irrigants and is characterized by dilutional hyponatremia [6,13]; it is not expected with 0.9% saline, but saline retained in the bowel remains available for absorption. At 24 hours, serum sodium had fallen from 138 to 131 mmol/L and chloride from 105 to 93 mmol/L, while bicarbonate rose from 23.7 mmol/L on admission to 27.4 mmol/L the following morning. Absorption of a substantial volume of saline, whose sodium and chloride concentrations both exceed those of plasma, would be expected to raise chloride and produce a hyperchloremic acidosis. The opposite occurred: chloride fell disproportionately to sodium and bicarbonate rose, a pattern more consistent with loss of chloride-rich gastric fluid through continuous nasogastric suction, superimposed on the mild hyponatremia common after surgery. There was therefore no biochemical evidence of clinically significant absorption of the irrigant, and the patient remained clinically well: he was awake and oriented on arrival in the intermediate care unit, lactate was 0.4 mmol/L, and the arterial blood gas had normalized by the following morning.

Recognition and hypothermia

Recognition was delayed because the abdomen and stoma appliance lay beneath the drapes, the lithotomy position and urological field restricted access, and the pouch and appliance filled at low pressure without producing tension. Detection therefore cannot be left to incidental observation: scheduled inspection of the abdomen and any stomal appliance, with measured comparison of irrigation input and drainage return, should form part of the anesthetic plan.

A temperature of 33.0 °C on admission to the intermediate care unit represents significant hypothermia, with recognized risks of coagulopathy, surgical site infection, and delayed recovery [15], and it occurred despite forced-air warming throughout; the irrigation fluid (20-22 °C) and intravenous fluids were unwarmed. Retained irrigation fluid acts as an internal heat sink: warming one liter from 21 °C to core temperature absorbs approximately 65 kJ, and at a whole-body heat capacity of about 3.5 kJ/kg/°C a 61 kg patient loses roughly 0.3 °C for each liter retained. The 750 mL recovered from the appliance and the 400 mL aspirated from the stomach represent only the fraction still accessible at the end of surgery. Unexplained or disproportionate hypothermia may therefore be an early and accessible signal of this complication.

Airway management

Selecting an airway device for this patient required balancing several considerations. The surgery was elective, the patient was appropriately fasted, the planned duration was within the range commonly managed with a supraglottic airway, and the lithotomy position is broadly compatible with supraglottic airway use [9,11]. However, the 4th National Audit Project specifically identified aspiration as the most common cause of airway-related deaths reported to that audit, and the use of supraglottic devices in patients with risk factors for regurgitation was a recurrent theme in the events analyzed [9,16]. An ileal pouch in continuity with the bladder under continuous irrigation is a risk factor that conventional aspiration assessment does not capture.

The most actionable lesson concerns gastric drainage: no orogastric tube was passed through the gastric channel of the i-gel. Although the i-gel is a second-generation supraglottic airway with a gastric drainage channel, its protective effect is contingent on correct positioning and on the assumption that regurgitated volumes fall within the drainage capacity of the device [16,17]. A tube in the channel would plausibly have decompressed the stomach, or given earlier warning through unexpected drainage of clear fluid, and we regard it as mandatory whenever a supraglottic device is used in a patient with a urinary-enteric communication. Tracheal intubation with a cuffed endotracheal tube would have provided an additional margin of safety in this setting. Recognizing that a single case cannot establish a standard, we offer as a considered recommendation, rather than as evidence-based guidance, that tracheal intubation be the default when such a fistula is known or suspected and continuous irrigation is planned, consistent with the principle that tracheal intubation should be used whenever there is a specific indication [18].

Aspiration, corticosteroids, and antimicrobial therapy

The intraoperative behavior of the regurgitated fluid was atypical for gastric content: it was clear, large in volume, and emerged in a pattern that coincided temporally with a marked reduction in cystoscopic irrigation return and with distension of the ileostomy appliance. Airway pressures, tidal volume, end-tidal carbo dioxide, oxygen saturation, and auscultation were unchanged; tracheal suction before ventilation was resumed yielded no gastric or particulate material; and the chest radiograph on the first postoperative day was clear. The material was nonetheless almost certainly a mixture of irrigation fluid and gastric secretions, and dilution of gastric acid may itself have limited pulmonary injury. A suction catheter samples only the trachea and proximal bronchi, and not every aspiration produces early clinical signs, so micro-aspiration cannot be excluded; the most that can be said is that there was no evidence of clinically significant aspiration. Because the two scenarios are not reliably separable at the moment of the event, conservative management - including oropharyngeal suctioning, conversion to a cuffed endotracheal tube, gastric decompression, and postoperative observation in a monitored environment - is appropriate and was undertaken in this case [19].

Neither corticosteroids nor antibiotics are indicated after witnessed aspiration without evidence of infection: corticosteroids have not been shown to improve outcome in aspiration pneumonitis, and early empirical antibiotics select resistant organisms without benefit [19]; fever alone in the first postoperative days would not have altered this. We report the hydrocortisone given when aspiration was suspected as it occurred; together with dexamethasone, it probably contributed to the leukocytosis through demargination. The rise in C-reactive protein to 280 mg/L, falling to 120 mg/L at 72 hours in an afebrile patient with negative cultures, is attributed to endoscopic trauma to a chronically contaminated, stone-bearing urinary tract, hypothermia, and the underlying fistulous disease. Perioperative prophylaxis in these patients should cover enteric flora, reflecting the urinary-enteric communication rather than aspiration risk.

Limitations

This is a single case, and the mechanism is inferred rather than demonstrated. Total irrigation input and cystostomy return were not measured, so the fluid-balance argument rests on the volumes recovered rather than on a complete balance. The regurgitated and stomal fluid was not analyzed biochemically, although sodium, chloride, osmolality, glucose, and pH would have distinguished irrigation saline from gastric content simply and inexpensively. Serum electrolytes were not paired with a simultaneous acid-base sample, and the time to normothermia was not recorded. Dedicated intraoperative imaging (for example, rapid cystographic evaluation with diluted contrast immediately after the event) could have provided additional anatomical confirmation but was not feasible in our setting. In any future suspected case, we would recommend measured input and returns, biochemical analysis of all recovered fluids, serial electrolytes, and photographic documentation of the distended appliance.

## Conclusions

Endoscopic urological procedures performed with continuous irrigation in patients with enterovesical fistulas carry a previously underrecognized risk of retrograde migration of irrigation fluid into the gastrointestinal tract, with subsequent regurgitation and potential pulmonary aspiration. On the basis of this single case, we offer the following as considered recommendations rather than established standards. Where a urinary-enteric fistula is known or suspected and continuous irrigation is planned, we favor tracheal intubation with a cuffed tube as the default airway strategy. Where a supraglottic device is nevertheless used, as will happen when the fistula is unsuspected, a second-generation device should be chosen and an orogastric tube passed through its gastric channel at induction and left on free drainage; this is the simplest measure likely to have altered the course of the present case.

Bag height should be set deliberately and documented, but height limitation alone does not prevent migration across a wide fistula. Flow should be intermittent rather than continuous where the procedure allows, bladder overdistension avoided, and the irrigation fluid warmed. The most reliable early warning is divergence between irrigation input and cystostomy or urethral return, which should be measured rather than estimated, with irrigation interrupted whenever the two diverge. Stoma appliances, catheters, and the drapes below a patent pouch-anal anastomosis should be inspected at defined intervals, and unexplained hypothermia should be treated as a sign of retained unwarmed fluid within the gut. Close multidisciplinary communication between urology, general surgery, and anesthesiology about the specifics of fistulous anatomy, previous reconstructions, and the surgical plan is essential for appropriate perioperative risk assessment in this patient population.
